# Biological and behavioral factors modify urinary arsenic metabolic profiles in a U.S. population

**DOI:** 10.1186/s12940-016-0144-x

**Published:** 2016-05-26

**Authors:** Edward E. Hudgens, Zuzana Drobna, Bin He, X. C. Le, Miroslav Styblo, John Rogers, David J. Thomas

**Affiliations:** Environmental Public Health Division, National Health and Environmental Effects Research Laboratory, Office of Research and Development, U.S. Environmental Protection Agency, Research Triangle Park, NC 27709 USA; Department of Nutrition, University of North Carolina at Chapel Hill, Chapel Hill, NC 27599 USA; Analytical and Environmental Toxicology, Department of Laboratory Medicine and Pathology, Faculty of Medicine and Dentistry, University of Alberta, Edmonton, AB T6G 2G3 Canada; Westat, 1600 Research Boulevard, Rockville, MD 20850 USA; Integrated Systems Toxicology Division, National Health and Environmental Effects Research Laboratory, Office of Research and Development, U.S. Environmental Protection Agency, Research Triangle Park, NC 27709 USA

**Keywords:** Inorganic arsenic, Methylated arsenicals, Drinking water, Urine, Toenails, Human, Biological and behavioral modifiers, Gender, Smoking, BMI

## Abstract

**Background:**

Because some adverse health effects associated with chronic arsenic exposure may be mediated by methylated arsenicals, interindividual variation in capacity to convert inorganic arsenic into mono- and di-methylated metabolites may be an important determinant of risk associated with exposure to this metalloid. Hence, identifying biological and behavioral factors that modify an individual’s capacity to methylate inorganic arsenic could provide insights into critical dose-response relations underlying adverse health effects.

**Methods:**

A total of 904 older adults (≥45 years old) in Churchill County, Nevada, who chronically used home tap water supplies containing up to 1850 μg of arsenic per liter provided urine and toenail samples for determination of total and speciated arsenic levels. Effects of biological factors (gender, age, body mass index) and behavioral factors (smoking, recent fish or shellfish consumption) on patterns of arsenicals in urine were evaluated with bivariate analyses and multivariate regression models.

**Results:**

Relative contributions of inorganic, mono-, and di-methylated arsenic to total speciated arsenic in urine were unchanged over the range of concentrations of arsenic in home tap water supplies used by study participants. Gender predicted both absolute and relative amounts of arsenicals in urine. Age predicted levels of inorganic arsenic in urine and body mass index predicted relative levels of mono- and di-methylated arsenic in urine. Smoking predicted both absolute and relative levels of arsenicals in urine. Multivariate regression models were developed for both absolute and relative levels of arsenicals in urine. Concentration of arsenic in home tap water and estimated water consumption were strongly predictive of levels of arsenicals in urine as were smoking, body mass index, and gender. Relative contributions of arsenicals to urinary arsenic were not consistently predicted by concentrations of arsenic in drinking water supplies but were more consistently predicted by gender, body mass index, age, and smoking.

**Conclusions:**

These findings suggest that analyses of dose-response relations in arsenic-exposed populations should account for biological and behavioral factors that modify levels of inorganic and methylated arsenicals in urine. Evidence of significant effects of these factors on arsenic metabolism may also support mode of action studies in appropriate experimental models.

**Electronic supplementary material:**

The online version of this article (doi:10.1186/s12940-016-0144-x) contains supplementary material, which is available to authorized users.

## Background

In a study population of older adults from Churchill County, Nevada, we have previously examined relations between dose (measured by the total arsenic (TAs) concentration in home tap water) and response (measured as summed urinary concentrations of speciated arsenicals) and modification of these relations by both biological (e.g., gender) and behavioral (e.g., smoking) factors [1]. Although informative, this analysis did not directly examine dose-response relations for individual urinary metabolites of inorganic arsenic (iAs) or effects of modifying factors on urinary levels of iAs and its monomethylated (MMA) and dimethylated (DMA) metabolites. These relations and their modification by biological and behavioral factors may be significant in assessing the consequences of chronic exposure to iAs. For example, methylation of iAs affects patterns of tissue distribution and facilitates the clearance of arsenic in humans and other species [2–5]. In humans, genotypic variation in *AS3MT* which encodes the enzyme that catalyzes reactions that methylate arsenic in humans has been linked to differences in urinary profiles of iAs and its methylated metabolites and to variation in disease susceptibility [6, 7]. Thus, characterizing effects of biological and behavioral factors on these dose-response relations may clarify their contributions to interindividual variation in capacity to convert iAs into methylated metabolites.

Churchill County, Nevada, presented a unique opportunity to evaluate the influence of these factors in a relatively large number of study participants (904) who were chronically exposed to TAs through home tap water supplies. Here, levels of TAs exposure in home tap water ranged from below the analytical limit of detection (LOD) to levels that have been consistently associated with health effects in other populations [8–10]. The present study characterized urinary excretion of iAs and its methylated metabolites in older adults who were chronically exposed to TAs through home tap water supplies. This study evaluated several biological factors (gender, age, body mass index (BMI) and behavioral factors (smoking, recent fish or shellfish consumption) as potential modifiers of urinary iAs, MMA, and DMA concentrations. These analyses found that all biological factors and a behavioral factor, smoking behavior, significantly affected absolute or relative levels of iAs or its methylated metabolites in urine. These modifying factors may contribute to variability among individuals in profiles of arsenicals in urine and in interindividual differences in susceptibility to adverse health effects associated with chronic exposure to iAs. Future studies of dose-response relations in exposed human populations should consider these modifiers on patterns of urinary arsenicals to assure accurate attributions of health effects.

## Methods

### Study design, sample collection and processing

Demographic characteristics of study participants are briefly summarized in the Additional file 1 and more fully elsewhere [1]. At the time of this study (August and September 2002), TAs concentration in the drinking water supply of Fallon, the largest city in Churchill County, exceeded the then-current maximum contaminant level (MCL) of 50 μg of TAs per liter [11]. Historically, TAs concentration in Fallon’s municipal water supply was around 100 μg per liter; during this study, the mean concentration was 89 μg per liter. To broaden the range of TAs exposures, participants includes users of the Fallon municipal water supply and individuals who resided elsewhere in Churchill County where TAs concentrations in home tap water supplies ranged from below the LOD (3 μg per liter) to 1850 μg per liter. Among 904 study participants, 250 used the municipal water supply, 613 used individual residential wells, and 41 used shared residential wells.

Criteria for enrollment in this study were an age of at least 45 years, continuous residency in Churchill County for 5 years at the time of enrollment, and cumulative residency of at least 20 years in the county. At enrollment, participants provided written consent to a study protocol approved by the Institutional Review Board of the University of North Carolina at Chapel Hill and reviewed by the U.S. EPA Human Subjects Protection Office.

Each participant provided a urine sample, a blood sample, and completed an exposure assessment and medical history questionnaire that requested demographic information, medical history, information on recent and habitual seafood consumption, length of residence in their present home, and any point-of-use treatment of home tap water or use of commercially bottled water. Participants reported daily water consumption, drug, alcohol, or tobacco usage, and potential environmental or occupational exposure to arsenic. Participants received a home collection kit for toenails and a container for an untreated home tap water sample. Home tap water samples were collected as described in Additional file 1. Urine samples were initially stored at 4 °C, shipped to North Carolina on dry ice, and stored at −80 °C until analyzed. Blood samples collected in EDTA-containing Vacutainer tubes (Becton-Dickson, Franklin Lakes, NJ) were initially stored at 4 °C, shipped to North Carolina on refrigerant packs, and then stored at 4 °C until processed.

### Total arsenic in drinking water

TAs concentrations in home tap water samples were determined by Environmental Protection Agency Method 200.8 [12] or by ASTM method D2972 [13] in the State Health Laboratory, Bureau of Health Protection Services, Reno, Nevada. This laboratory, accredited by US EPA Region IX, used quality assurance and quality control procedures that met Safe Drinking Water Act guidelines.

### Speciated arsenicals in urine

Urinary concentrations of arsenite (iAs^III^) arsenate (iAs^V^), monomethylarsonic acid (MMA^V^) and dimethylarsinic acid (DMA^V^) were determined by ion-pair chromatographic separation with hydride generation-atomic fluorescence detection [14] as described in Additional file 1. Concentrations of these urinary metabolites were expressed on a parts per billion basis without correction for urinary creatinine concentration or specific gravity. Limits of detection for iAs^III^ and MAs^V^ were 0.5 μg/l; for iAs^V^ and DMAs^V^, 1 μg/l.

### Toenail total arsenic

Total arsenic concentrations in toenail samples (NTAs) were determined by instrumental neutron activation analysis and expressed as parts per million of arsenic [1]. Sample collection, processing, and analysis are described in Additional file 1.

### Urinary cotinine and creatinine

Urinary cotinine levels were used to assess cigarette smoking. Urinary cotinine concentrations were determined by radioimmunoassay [15, 16]. Except for creating a smoking/non-smoking categorical variable from creatinine-corrected urinary cotinine concentrations that was used for the stepwise selection, urinary concentrations of arsenicals and cotinine were not corrected for creatinine concentration. Methods for determination of cotinine and creatinine in urine are described in Additional file 1.

### Imputation of non-detects and missing values

Samples with the concentration of an analyte below the LOD were designated as non-detect samples. Concentrations in non-detect samples and other missing values were imputed for the analysis. Imputation of analyte concentrations in non-detect samples assumed that analyte concentrations were log-normally distributed and the distribution of concentrations in the non-detect samples resembled the portion of the log-normal distribution below the detection limit. In 21 tap water samples, TAs was below the LOD and in 52 toenail samples NTAs was below the LOD. Among urine samples, iAs^III^ and iAs^V^ were not detected in 289 and 583 samples, respectively, MMA^V^ was not detected in 217 samples, and DMA^V^ was not detected in 46 samples. Besides imputation of values for non-detect samples, some samples were missing. For example, 59 participants did not provide toenail samples; one participant did not provide urine samples for cotinine and creatinine determinations or data needed for calculation of BMI.

All non-detect and missing values were imputed in a manner consistent with the cause of the missing value and that maintained correlations among analysis variables. Because imputation has a random component, results from analysis of imputed data also have a random component. To minimize the magnitude of the random component and to calculate standard errors and confidence intervals that reflect uncertainty in imputed values, multiple imputations were performed. This procedure yielded 20 imputed data sets for analysis [17]. The imputation process is further described in Additional file 1.

### Calculation of analysis variables

The analysis used imputed TiAs, MMA and DMA concentrations or derived quantities. Urinary iAs^III^ and iAs^V^ concentrations were summed for a total iAs concentration term (TiAs). Urinary TiAs, MMA and DMA concentrations were summed to create a urinary speciated arsenic concentration term (USAs). Urinary %TiAs, %MMA, and %DMA were calculated using USAs as the denominator. A primary methylation index (PMI) was calculated as the MMA^V^:TiAs concentration ratio and a secondary methylation index (SMI) was calculated as the DMA^V^:MMA^V^ concentration ratio.

Continuous variables were positively skewed and log_10_-transformed values were roughly symmetrically distributed. To yield approximately normally distributed regression residuals with constant variance, absolute urinary concentration variables were log_10_-transformed and relative urinary concentrations were logit transformed. Continuous predictor variables were log_10_-transformed to make their distributions more normally distributed and to minimize the influence of particularly large values. Before log-transforming reported tap water consumption, 93 zeroes were recoded to 0.06 l per day (one-quarter cup). Unless otherwise noted, categorical variables were recoded as dummy variables with the reference category coded as zero.

### Bivariate statistical analysis using imputed data

All analysis and data processing were performed with SAS version 9.4. The 95 % significance level was used for assessing significant results. For bivariate analyses, the SAS MIXED procedure was used to estimate means and correlations and to perform t-tests. The analysis procedures were applied to all 20 imputed data sets. The 20 sets of parameter results were passed through the MIANALYZE procedure to calculate final parameter estimates, standard errors, and p-values adjusted for the imputation process. Percentiles were calculated using all imputed data sets; plots show data from the first imputed data set.

### Comparison of urinary arsenic concentrations in study participants and NHANES

We compared the distribution of levels of arsenicals in urine of Churchill County study participants with the distribution of these levels calculated from nationally representative 2003–2012 NHANES data [18]. Here, we calculated mean log_10_-transformed concentrations of urinary arsenic species for demographic groups that were used in analysis of Churchill county data (gender, race (white *vs* non-white), and age (40 to 49, 50 to 59, 60 to 69, 70 to 79, and 80 years or older). Means were calculated using survival analysis (using the LIFEREG procedure with the NHANES survey weights) to adjust for non-detects. NHANES data was then reweighted to represent the demographic distribution of the Churchill County study population, using the demographic groups identified above. Mean log_10_-transformed urinary arsenical concentrations (predicted from survival analysis), mean detection limit for non-detects, and percentage of non-detects were calculated using revised weights. Resulting values were used to estimate the NHANES geometric mean concentrations of urinary arsenical species, mean detection limits, and percentage of non-detects for a population similar to the Churchill County study population. These values were compared to similar statistics calculated from Churchill County study data using imputed concentrations and indicators of non-detects. Comparative data for the Churchill County study and the NHANES survey data are summarized in Additional file 1.

### Multivariate statistical analysis using imputed data

Given the large number of variables in the data set, a procedure was needed to select independent variables used to model urinaary arsenic-dependent variables. This analysis made two assumptions. First, variables that were significant when modeling one dependent variable might be important for predicting other dependent variables. Second, analysis using nine different but related urinary arsenic-dependent variables would identify the most relevant set of independent variables for modeling.

This analysis used multiple imputation to provide estimates of missing values of variables needed for model development. As described in detail in Additional file 1, this approach provided plausible substitute values for missing data and allowed estimation of standard errors that included uncertainty due to the imputation process. A stepwise selection procedure identified candidate variables for prediction of nine dependent variables that are biomarkers of arsenic exposure (log_10_-transformed urinary TiAs, MMA, DMA, and USAs concentrations, PMI, SMI, and logit-transformed urinary %TiAs, %MMA, %DMA). Candidate variables included biological factors (gender, race (white versus other), BMI, age, and urinary creatinine concentration), behavioral factors (smoking, recent alcohol consumption, and fish and seafood consumption) and characteristics of water consumption (TAs concentration in home tap water (as a linear and quadratic term), primary home water source, and daily water consumption). All continuous predictors were log_10_-transformed. Standard procedures for stepwise selection of linear regression predictors from a set of candidate predictors were adapted for use with multiple imputations. For 180 combinations of nine dependent variables and 20 imputed data sets, the SAS GLMSELECT procedure was applied with default selection criteria, with the exception that interactions were considered only if the associated main effects were already in the model. A candidate predictor that was selected in more than 25 of 180 models was retained for use in the final model. All candidate variables are listed in the Additional file 1.

The list of selected predictors was reviewed and revised as follows. Similar smoking-related predictors (a categorical smoking variable and log_10_-transformed urinary cotinine concentration) were selected in different models, necessitating choice of a variable for final model. Log_10_-transformed urinary cotinine concentration was used in final model development because it was selected in more imputed data sets and predicted more dependent variables than did the categorical smoking variable. This process selected various combinations of TAs and predictors related to water source and water treatment. These combinations were replaced by the interaction of drinking water source and TAs. Here, drinking water sources were divided into four self-reported categories: untreated tap water that was used without in-home treatment, filtered tap water subjected to in-home filtration, tap water that received other in-home treatment (e.g., reverse osmosis), or use of commercially bottled water as in-home water source. Predictive models were developed for the nine dependent variables, fitting separate intercepts and slopes for each of the four drinking water source categories (untreated tap water, filtered tap water, other treatment tap water, or commercially bottled water).

The stepwise procedure did not select an indicator of fish and shellfish consumption in the previous 48 h as a predictor (it was selected in 9 of the 180 combinations of dependent variables and imputed data sets). Because fish and shellfish consumption had been identified as a significant predictor of urine arsenic concentrations in other research [19], the indicator of recent fish and shellfish consumption was included in the final model.

### Non-linear term for TAs concentration

The stepwise regression procedure selected a quadratic relationship between the dependent variables and the log_10_-transformed home tap water TAs concentrations. Analysis of relations among log_10_-transformed water consumption, urinary arsenical concentrations, urinary creatinine concentration, and home tap water TAs concentrations indicated the following monotonic transformation of home tap water TAs concentrations provided a better fit and accounted for the apparent quadratic relationship,

$$ Lo{g}_{10}\left({\beta}_{Offset}+ WaterAs\right) $$

where *β*_*Offset*_ is a positive concentration offset. This non-linear term defines a monotonic relationship between water arsenic concentrations and urinary arsenic concentrations. The use of an offset term may represent contributions of sources of arsenic that were not otherwise represented in the model. Additional details are provided in Additional file 1.

### Modeling effects of biological and behavioral factors and exposure characteristics on urinary arsenical levels

Because the dependent variables were closely related, we posited that a variable that was important for prediction of one dependent variable would likely to be important for prediction of another dependent variable, although this contribution may not be statistically significant in all models. As a result, the same final model was fitted to all independent variables. The offset parameter for water arsenic concentration (*β*_*Offset*_) was modeled using the log_10_-transformed offset (*α*_*LogOffset*_) to avoid negative offset values and improve convergence.

The final model used to predict log-transformed biomarkers of exposure to arsenic (Y) is described by the following equation:

$$ \begin{array}{c}Lo{g}_{10}(Y)={\alpha}_{DWS}+{\beta}_{DWS}\left(Lo{g}_{10}\left({10}^{\alpha_{LogOffset}}+ WaterAs\right)\right)+{\beta}_QLo{g}_{10}\left( Tap\kern.5em  water\kern.5em  consumption\right)\\ {}\kern3em +{\beta}_CLo{g}_{10}(Creatinine)+{\beta}_F Female+{\beta}_BLo{g}_{10}(BMI)+{\beta}_ALo{g}_{10}(Age)\\ {}\kern3em +{\beta}_SLo{g}_{10}(Cotinine)+{\beta}_RLo{g}_{10}\kern.5em \left( Recent\kern.5em  fish\kern.5em  consumption\right).\end{array} $$

In this analysis, separate intercept (*α*_*DWS*_) and slope (*β*_*DWS*_) parameters were fitted for the four drinking water source categories. Although urinary concentrations of either arsenicals or cotinine were not corrected for creatinine concentration, creatinine was included in the multivariate model as a predictor. As a result, the parameters in the multivariate model represent the relationship between concentrations after adjusting for any effect of creatinine on the measurements. Tap water consumption is the self-estimated daily amount to tap water consumed, whether treated or untreated. For this analysis, recent fish or shellfish consumption was defined as self-reported consumption during the previous 48 h. It was scored as 1 if the subject participant reported consumption or as 0 if consumption did not occur. This non-linear model was fitted to each of the 20 imputed datasets using the SAS NLIN procedure. The 20 sets of parameter results were passed through the MIANALYZE procedure to calculate final parameter estimates, standard errors, and *p*-values adjusted for the imputation process.

## Results

**Fig. 1:**
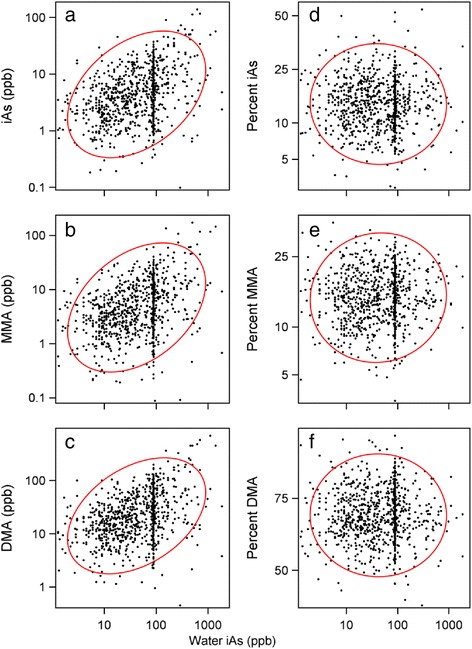
Relations between arsenic concentrations in home tap water and absolute or relative concentrations of arsenicals in urine. Panels **a**, **b**, and **c** show relations between home tap water arsenic concentration and urinary concentration of (**a**) inorganic arsenic, iAs, (**b**) monomethylated arsenic, MMA, and (**c**) dimethylated arsenic, DMA. Arsenic concentrations in home tap water and in urine expressed as parts per billion (ppb, μg per l). For each arsenical, there was a statistically significant correlation between arsenic levels in home tap water and in urine. Panels **d**, **e**, and **f** show relations between home tap water arsenic concentration and percentage of urinary speciated arsenic concentration accounted for by **d** iAs, **e** MMA, and (**f**) DMA. The red line in each panel encloses 95 % of data

**Fig. 2:**
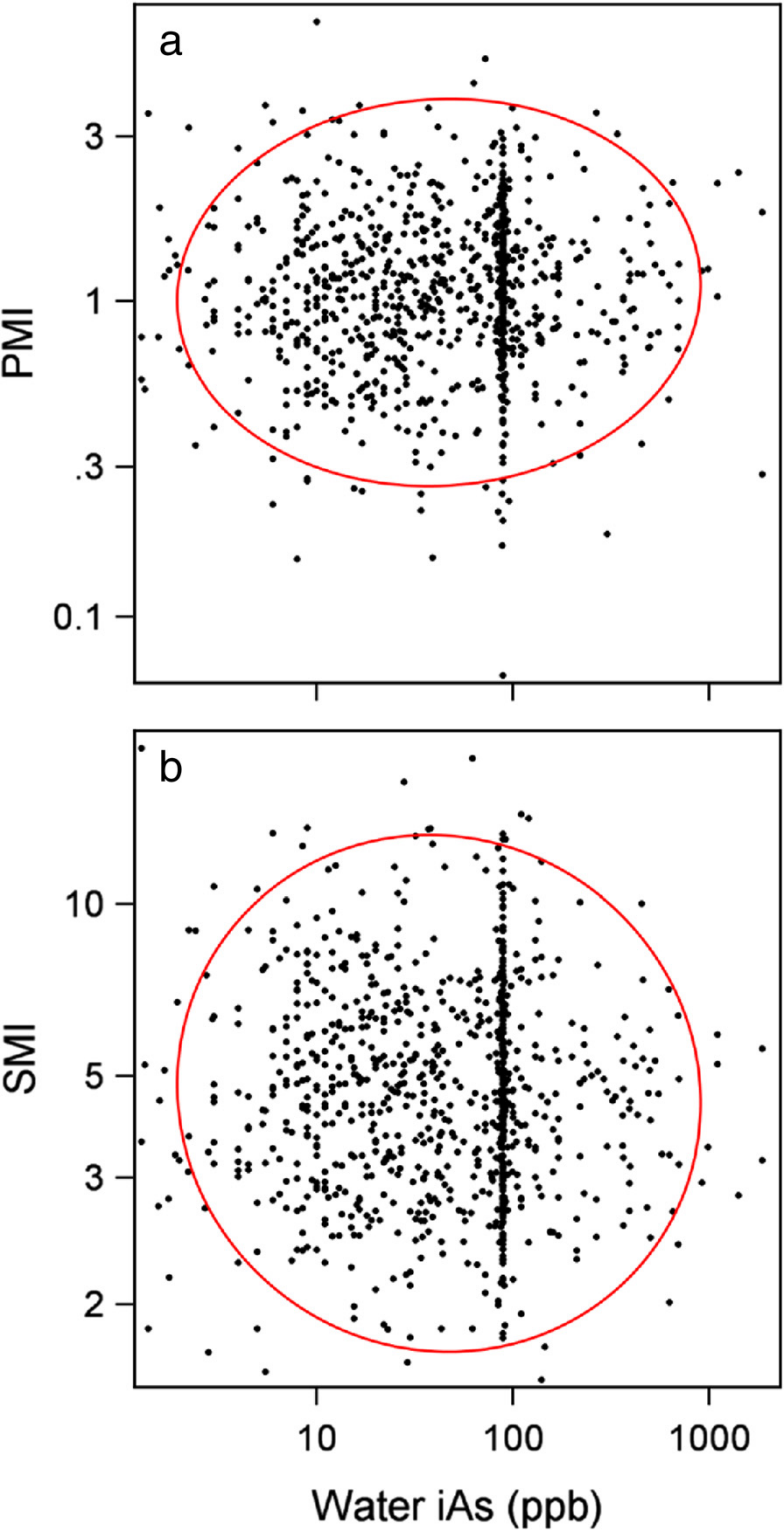
Relations between arsenic concentrations in home tap water and primary and secondary methylation indices. Panel **a** shows the relation between home tap water arsenic levels and the primary methylation index. Arsenic concentrations in home tap water expressed as parts per billion (ppb, μg per l). Panel **b** shows the relation between home tap water arsenic levels and the secondary methylation index. The red line in each panel encloses 95 % of data

Of 904 study participants, 59 % were female, most (84 %) were non-smokers, and 55 % of participants were 60 years of age or older. A full account of the characteristics of study participants has been presented [1]. For all study participants, home tap water TAs concentrations were used as the measure of exposure. Figure 1 shows urinary log_10_-transformed TiAs, MMA, and DMA concentrations and logit-transformed %TiAs, %MMA, and %DMA as functions of log_10_-transformed home tap water TAs concentrations. Increasing home tap water TAs concentrations were significantly associated with increasing urinary TiAs, MMA, and DMA concentrations. In contrast, urinary %TiAs, %MMA, and %DMA which varied widely among participants were not significantly correlated with home tap water TAs concentration. Based on the 1st and 99th percentiles, %TiAs ranged from 4.4 to 37.9 %, %MMA from 5.9 to 29.3 %, and %DMA from 47.4 to 86.0 %. Figure 2 shows the relation of PMI and SMI values to log_10_-transformed home tap water TAs concentrations. Based on the 1st and 99th percentiles, PMI ranged from 0.22 to 4.02 and SMI ranged from 1.81 to 13.49. Neither methylation index significantly correlated with home tap water TAs concentration.

Notably, among study participants, concentrations of some urinary analytes were below LODs. The species with the highest rates of non-detection were iAs^V^ (64 % of urine samples) and iAs^III^ (32 % of urine samples). MMA was not detected in 24 % of urine samples and DMA was not detected in 5 % of urine samples. Absence of quantifiable levels of these metabolites in urine prompted the imputation of values used in data modeling.

To evaluate the significance of non-detect levels of arsenicals in urine, we compared distributions of urinary concentrations of arsenicals and frequencies of non-detect levels in study participants with corresponding values calculated with 2003–2012 NHANES survey data. As summarized in Additional file 1, geometric mean urinary concentrations of MMA, and DMA for study participants were 5.6 to 7.9 times higher than corresponding values calculated from NHANES survey data. Similarly, percentages of non-detect values for each arsenical in urine were much higher in NHANES survey than in study participants.

**Fig. 3:**
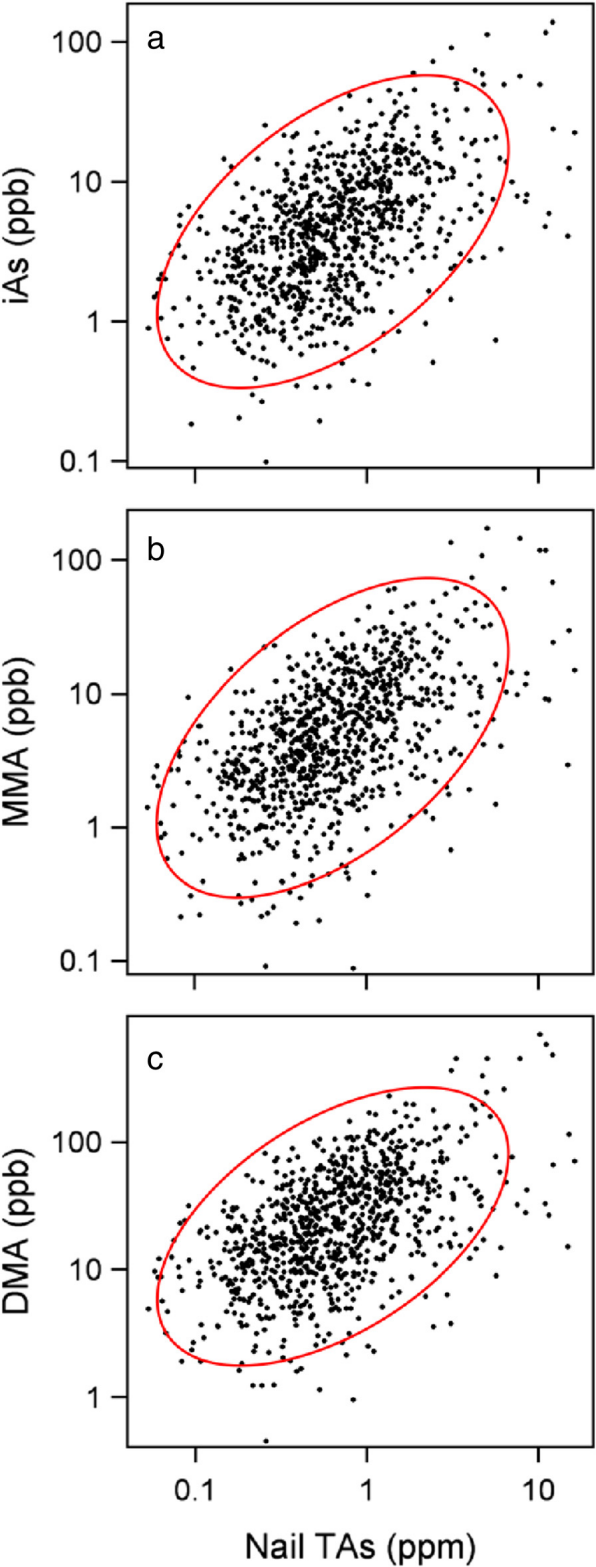
Relation between concentrations of total arsenic in nails and concentrations of inorganic arsenic and its methylated metabolites in urine, **a** iAs, **b** MMA, and **c** DMA. Arsenic concentrations expressed as parts per million (ppm, μg per g) in nails and as parts per billion (ppb, μg per l) in urine

Relations between NTAs and urinary TiAs, MMA, or DMA concentrations were examined (Fig. 3). Over the 100-fold range of NTAs concentrations, there were a significant positive correlations (*P* < 0.0001) between NTAs concentrations and urinary concentrations of each arsenical.

Gender significantly correlated with absolute and relative concentrations of TiAs and its methylated metabolites in urine (Table 1). Compared to males, females had significantly lower urinary concentrations of TiAs, MMA, and DMA (*P* ≤ 0.0009), %TiAs (*P* = 0.0046) and %MMA (*P* < 0.0001). In contrast, urinary %DMA in females was significantly higher than in males (*P* < 0.0001). The geometric mean PMI was not significantly correlated with gender; however, the geometric mean SMI was significantly higher (*P* < 0.0001) in females than in males.

**Table 1:** Effects of gender on absolute and relative levels of arsenicals in urine

Arsenical		Gender		*P*-value^a^
		Male	Female	
TiAs	Geometric mean (μg/l)	2.070	1.737	<.0001
	Mean %	15.7	14.1	0.0046
MMA	Geometric mean (μg/l)	2.204	1.789	<.0001
	Mean %	17.3	14.5	<.0001
DMA	Geometric mean (μg/l)	4.041	3.661	0.0009
	Mean %	67.1	71.4	<.0001
Methylation Index (Geometric mean)				
Primary		1.065	1.030	0.1308
Secondary		1.834	2.046	<.0001

^a^ *P*-values for the geometric mean differences are based on analysis of the log_10_-transformed values. *P*-values for mean percentage differences are based on analysis of the logit transformed percentages

Enrollment criteria for this study yielded a sample in which 45 % of participants were from 45 to 59 years old and 55 % from 60 to 92 years old. The median age of study participants was 61 years. There was a significant age-dependent trend for lower urinary TiAs concentration (*P* = 0.0072). Although urinary MMA and DMA concentrations declined with increasing age, these trends were not statistically significant. Increasing age significantly correlated with PMI (*P* = 0.0002) but not with SMI. There were significant age-related trends for decreased %TiAs (*P* < 0.0001) and increased %DMA (*P* < 0.0029) in urine.

No statistically significant relations were found between BMI and urinary TiAs, MMA, or DMA concentrations. Increased BMI was associated with significantly decreased %MMA (*P* < .0001), significantly increased %DMA (*P* = 0.0002), significantly lower PMI (*P* = 0.0013), and significantly higher SMI (*P* < 0.0001).

Smoking frequency, estimated by urinary cotinine concentration, was associated with significantly increased urinary TiAs, MMA, and DMA concentrations (*P* ≤ 0.0001), and urinary %TiAs (*P* = 0.0013), and %MMA (*P* = 0.0185), but with decreased urinary %DMA (*P* < 0.0001). Smoking frequency was not associated with PMI but was associated with significantly decreased SMI (*P* = 0.0016).

The questionnaire completed by study participants collected information on recent (i.e., within 48 h) consumption of fish or shellfish. The current analysis examined whether recent consumption of fish or shellfish affected absolute or relative levels of inorganic or methylated arsenicals in urine. In this study population, no significant bivariate relations were seen between intake of these seafoods and levels of speciated arsenicals in urine.

**Fig. 4:**
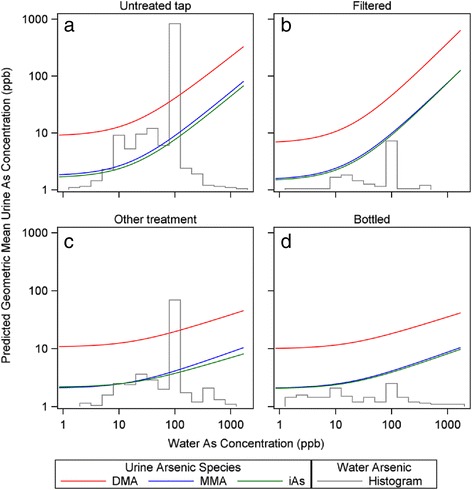
Concentrations of inorganic arsenic and its methylated metabolites in urine predicted by multivariate regression model. Levels of inorganic arsenic (iAs), monomethylated arsenic (MMA), and dimethylated arsenic (DMA) in urine predicted for users of home tap water that is untreated **a**, filtered **b**, or receives other treatment **c** and users of commercially bottled water **d**. Histogram (gray line) shows distribution of home tap water TAs concentrations for each user group. Arsenic concentrations in home tap water and in urine expressed as parts per billion (ppb, μg per l)

The final model predicted log_10_-transformed urinary TiAs, MMA, DMA, USAs concentrations, logit-transformed urinary %TiAs, %MMA, and %DMA, and log_10_-transformed methylation indices, PMI and SMI. Parameter estimates for the model are shown in Tables 2 and 3. Because the same model was fitted to all dependent variables that are biomarkers of exposure to arsenic, parameter estimates can be compared across models. Figure 4 shows predicted relations between home tap water TAs concentrations and urinary TiAs, MMA, and DMA concentrations for each water use group and includes a histogram of home TAs concentrations for each drinking water source category. Predicted relationships shown in Fig. 4 were calculated by setting values for gender and all log_10_-transformed continuous variables except home water TAs concentrations to their mean across all respondents.

**Table 2:** Model parameter estimates of concentrations of arsenicals in urine^a^

Parameter		Arsenical			
		Log_10_ (TiAs)	Log_10_ (MMA)	Log_10_ (DMA)	Log_10 _(USAs)
Intercept	Untreated tap	−1.068* (0.504)	−1.337* (0.453)	−1.70** (0.382)	−1.208* (0.382)
	Filtered	−1.328* (0.574)	−1.561* (0.506)	−2.068** (0.439)	−1.529*** (0.436)
	Other treatment	−0.382 (0.448)	−0.703 (0.410)	−1.077* (0.344)	−0.568 (0.344)
	Bottled	−0.291 (0.447)	−0.699 (0.405)	−1.05* (0.339)	−0.529 0.338)
Transformed Water TAs Slope	Untreated tap	0.813** (0.121)	0.817** (0.105)	0.776** (0.091)	0.791** (0.091)
	Filtered	0.978** (0.177)	0.945** (0.152)	0.978** (0.138)	0.969** (0.136)
	Other treatment	0.342*** (0.088)	0.346** (0.081)	0.305** (0.067)	0.317** (0.068)
	Bottled	0.289*** (0.074)	0.345** (0.065)	0.309** (0.055)	0.306** (0.055)
Log_10_ (Water TAs offset)		1.244** (0.220)	1.20** (0.194)	1.206** (0.175)	1.212** (0.172)
Log_10_ (Tap water consumption)		0.102* (0.033)	0.139** (0.029)	0.136** (0.024)	0.130** (0.024)
Log_10_ (Creatinine)		0.580** (0.046)	0.714** (0.041)	0.732** (0.033)	0.706** (0.033)
Gender (Female = 1, Male = 0)		−0.049 (0.027)	−0.061* (0.024)	0.056* (0.020)	0.023 (0.020)
Log_10_ (BMI)		−0.147 (0.156)	−0.628* (0.140)	−0.072 (0.118)	−0.164 (0.117)
Log_10_ (Age)		−0.360* (0.169)	0.101 (0.150)	0.211 (0.129)	0.118 (0.129)
Log_10_ (Cotinine)		0.054** (0.012)	0.031* (0.011)	0.023* (0.009)	0.029* (0.009)
Fish or shellfish consumption within 48 h		0.030 (0.028)	0.011 (0.025)	0.039 (0.021)	0.034 (0.021)

^a^Parameter estimates with standard errors in parentheses. Significance levels for estimates: *P* < 0.0001-***; 0.0001 < *P* < 0.001-**; 0.05 > *P* > 0.001-*

**Table 3:** Model parameter estimates of relative urinary arsenic levels and methylation indices^a^

Parameter		Arsenical			Methylation index	
		Logit (%TiAs)	Logit (%MMA)	Logit (%DMA)	Log_10_ (PMI)	Log_10_ (SMI)
Intercept	Untreated tap	0.969 (0.689)	0.178 (0.496)	−1.867** (0.459)	−0.311 (0.348)	−0.387 (0.214)
	Filtered	1.136 (0.711)	0.369 (0.509)	−2.114** (0.479)	−0.295 (0.353)	−0.492* (0.221)
	Other treatment	1.047 (0.676)	0.239 (0.489)	−1.962** (0.458)	−0.315 (0.339)	−0.424* (0.213)
	Bottled	1.138 (0.700)	0.113 (0.500)	−1.965** (0.459)	−0.387 (0.355)	−0.380 (0.215)
Transformed Water TAs Slope	Untreated tap	0.012 (0.063)	0.062 (0.042)	−0.053 (0.041)	0.020 (0.031)	−0.031 (0.018)
	Filtered	−0.033 (0.128)	−0.049 (0.087)	0.065 (0.085)	−0.009 (0.061)	0.028 (0.038)
	Other treatment	0.045 (0.073)	0.024 (0.055)	−0.047 (0.053)	−0.006 (0.035)	−0.015 (0.024)
	Bottled	−0.063 (0.073)	0.070 (0.052)	0.011 (0.053)	0.046 (0.034)	−0.023 (0.023)
Log_10_ (Water TAs offset)						
Log_10_ (Tap water consumption)		−0.079 (0.055)	0.023 (0.039)	0.039 (0.038)	0.037 (0.027)	−0.003 (0.017)
Log_10_ (Creatinine)		−0.341** (0.078)	0.019 (0.054)	0.193*** (0.051)	0.133* (0.039)	0.018 (0.023)
Gender (Female = 1, Male = 0)		−0.195** (0.046)	−0.226** (0.031)	0.257** (0.029)	−0.012 (0.022)	0.117** (0.013)
Log_10_ (BMI)		0.055 (0.270)	−1.257** (0.183)	0.736** (0.173)	−0.478*** (0.133)	0.554** (0.079)
Log_10_ (Age)		−1.277** (0.272)	−0.040 (0.189)	0.719*** (0.188)	0.461*** (0.131)	0.110 (0.084)
Log_10_ (Cotinine)		0.067*** (0.019)	0.007 (0.014)	−0.046*** (0.014)	−0.023* (0.009)	−0.008 (0.006)
Fish or shellfish consumption within 48 h		−0.011 (0.047)	−0.061 (0.032)	0.040 (0.032)	−0.019 (0.023)	0.028 (0.014)

^a^Parameter estimates with standard errors in parentheses. Significance levels for estimates: *P* < 0.0001-***; 0.0001 < *P* < 0.001-**; 0.05 > *P* > 0.001-*

Many parameters, particularly slope parameters for transformed TAs concentration in the drinking water source, significantly predicted urinary biomarkers of arsenic exposure. As home tap water TAs concentrations and tap water consumption increased, urinary concentrations of arsenicals increased, other factors being constant. Higher urinary concentrations of arsenicals were associated with use of untreated tap water or filtered tap water compared to use of other treatment tap water or bottled water. Smoking frequency as reflected by urinary cotinine concentration increased urinary concentrations of arsenicals, especially TiAs. Increasing BMI was associated with lower urinary arsenical concentrations, although this relationship was statistically significant for only MMA. Influences of age and gender on urinary arsenical concentrations were less significant and inconsistent in direction among different dependent variables. Self-reported recent consumption of fish or shellfish consumption was not a significant predictor of any of the arsenic-dependent variables.

A similar approach was taken to analyze statistical significance of parameter estimates in prediction of relative urinary arsenical concentrations or of methylation indices (Table 3). Neither transformed TAs concentration nor tap water consumption significantly predicted these dependent variables. Differences in intercepts or slopes were not statistically significant among drinking water sources. When log_10_-transformed TAs concentration was not a significant predictor, the offset parameter was also not significant or important to the model. This observation, coupled with difficulties in estimating the offset, lead to its removal from the final model for predicting relative concentrations. Females and older respondents had relatively less arsenic as iAs and MMA and more as DMA. Those with higher BMI had relatively less arsenic as MMA and more as DMA. Higher urinary cotinine concentrations were associated with higher levels of iAs in urine.

## Discussion

In the following sections, the influence of biological and behavioral factors on conversion of iAs into methylated metabolites are considered singly and as they contribute to a multivariate regression model that characterized significant factors associated with interindividual metabolic capacity.

As in the earlier analysis of data from study participants [1], the current analyses used urinary concentrations of arsenicals that were not corrected for creatinine concentration. Urinary creatinine concentration is related to age [20, 21]. An earlier study found that creatinine correction of arsenic concentrations in urine did not improve exposure estimates [22] and adjustment for urinary creatinine concentration resulted in overestimation of urinary arsenic levels in individuals with type 2 diabetes [23]. Notably, our earlier analysis found urinary creatinine concentration to be a significant predictor of the sum of concentrations of speciated arsenicals in study participants [1]. This relation likely reflects interactions between creatinine metabolism and the enzymatically catalyzed reactions that methylate arsenic [24, 25] as well as factors affecting urine dilution.

The relation between extent of exposure to arsenic as exemplified by TAs concentrations in home tap water supplies and urinary TiAs, MMA, and DMA concentrations was examined in study participants with home TAs concentrations up to 1,850 μg per liter. Over this exposure range, there were significant correlations between concentrations of TAs in home tap water supplies and urinary TiAs, MMA, and DMA concentrations. These correlations were also found in a Bengladeshi population with a similar range of concentrations of TAs in drinking water supplies [26]. In contrast, relative levels of TiAs, MMA, and DMA in urine were not correlated with home tap water TAs concentrations. A lack of correlation between concentration of arsenic in drinking water and relative levels of urinary arsenicals has been previously reported [27]. The relation between intensity of exposure to iAs and the relative levels of inorganic and methylated arsenicals in urine can be evaluated as a reflection of the characteristics of reactions that methylate arsenic. These reactions are catalyzed by arsenic (+3 oxidation state) methyltransferase (AS3MT) and require S-adenosylmethionine (AdoMet) as the methyl group donor [28]. Upregulated *AS3MT* gene expression or AdoMet availability may be major determinants of capacity for arsenic methylation [29, 30]. Capacity for AS3MT-catalyzed conversion of iAs into methylated metabolites is apparently quite high. In workers exposed to arsenic fume and dust with estimated daily respired doses of arsenic up to 4000 μg, neither absolute nor relative iAs, MMA, and DMA concentrations in urine showed evidence of saturation [31]. However, under some situations, the capacity for enzymatically catalyzed methylation of arsenic may be saturated. In an iAs-exposed Bengaldeshi population, insufficient dietary intake of folic acid or folate and cobalamin affected folate- and cobalamin-dependent one-carbon metabolism, diminished AdoMet availability, and limited AS3MT-catalyzed conversion of iAs into its methylated metabolites [30]. Although neither folate nor cobalamin nutriture was evaluated in the current study, evidence from contemporaneous NHANES surveys suggest that among study participants folic acid/folate intake was likely sufficient [32] and cobalamin deficiency was probably uncommon [33]. In addition to the biological and behavioral factors considered in this study, polymorphisms of *AS3MT* in humans that alter catalytic properties of the methyltransfersase [34] can contribute to interindividual differences in urinary profiles of iAs and its methylated metabolites [7]. Although a comprehensive study of the role of *AS3MT* genotypic variation was beyond the scope of the current study, a pilot study summarized in Additional file 1 demonstrated that common *AS3MT* polymorphisms affected methylation capacity in study participants.

This study found no significant relationship between home tap water TAs concentration and either methylation index. Methylation indices are phenotypic indicators of arsenic methylation ability [35] and are widely used as stratifiers in population-based studies of adverse health effects associated with chronic arsenic exposure (e.g., [36, 37]). The ratios PMI and SMI are dimensionless quantities that may not reflect exposure-dependent changes in arsenic methylation efficiency. Furthermore, evaluation of urinary levels of speciated arsenicals from 2009–2010 NHANES survey data found seafood consumption can increase DMA intake [38]. Because DMA derived from seafood does not originate by conversion of iAs to methylated metabolites, its contribution to urinary DMA could affect the calculation of the SMI by increasing the denominator. Variation in PMI values (over 18-fold) and SMI values (over seven-fold) may reflect uncharacterized sources of exposure to arsenicals and confound the relation between exposure to iAs from home tap water supplies and metabolic indices.

Increased urinary TiAs, MMA, and DMA concentrations were associated with increasing NTAs concentrations. This finding is consistent with reports of strong correlations between NTAs concentration and either USAs concentrations or summed concentrations of all arsenicals in urine [1, 39]. NTAs concentrations have been positively associated with increasing exposure to arsenic from drinking water and food [40–43], although this relation can be modified by nutritional status or genotype [39, 40, 44]. The relation between urinary concentrations of speciated arsenicals and forms and concentrations of arsenic in nails remains unclear. Arsenic is concentrated in dorsal and ventral surfaces of nails in association with sulfur-rich molecules, especially keratin [45]. More than 85 % of arsenic in aqueous extracts of nails is iAs; DMA accounts for the balance [46]. Thus, although arsenic retained in nails may integrate exposure over longer timeframe than does arsenic in urine, it is unclear whether nails are useful biomarkers to reconstruct patterns of exposure to inorganic and methylated arsenicals [47, 48].

Among study participants, males reported significantly higher home tap water and total water consumption than did females [1]. This gender difference in water intake was reflected in higher urinary TiAs, MMA, and DMA concentrations in males than in females. Urinary %TiAs, %MMA, and %DMA also differed in males and females. Consistent with this study, a gender difference in urinary excretion of methylated arsenicals was found in a Bangladeshi population where the mean SMI in females was significantly higher than in males [49]. This between-gender difference in methylation efficiency was attributed to the influence of sex hormones. Because the median age of study participants (61 years) was higher than the median age at menopause (52.54 years) in the U.S. [50], it is unclear whether menopause per se was a factor in the gender difference in methylation capacity. Notably, between-gender differences in absolute and relative urinary concentrations of methylated arsenicals, particularly MMA, has been linked to gender differences in the prevalence of adverse health effects associated with chronic exposure to iAs [51].

Age-dependent trends for lower urinary TiAs, MMA, and higher DMA concentrations were reflected in altered relative urinary arsenical levels. Notably, our earlier analysis of age-stratified data found no statistically significant trends for USAs concentrations among study participants [1]. Median BMIs for male and female study participants were 28.0 and 28.8, respectively. These values are comparable with NHANES 1999–2002 results [52] for males (mean BMI by decade of 26.8 to 28.7) and females (mean BMI by decade of 26.8 to 29.2). Among study participants, increasing BMI was not correlated with urinary TiAs, MMA, and DMA concentrations but was associated with decreased urinary %MMA and increased %DMA. Notably, a reported association between increased BMI and lower levels of arsenic in nails [53] suggests that obesity may alter arsenical distribution and retention. Consistent with this study, associations between increased BMI and decreased urinary %MMA or increased %DMA were found in the Strong Heart Study cohort of adult residents of rural U.S. midwestern and southwestern communities [54] and in a cohort of adult women from the U.S. southwest and northwest Mexico [55, 56]. An interaction between elevated BMI and cancer risk in iAs-exposed adults has recently been described in a South American population [57].

Cigarette smoking was associated with increased absolute and relative urinary TiAs, MMA, and DMA concentrations and altered methylation indices. The contribution of arsenic in tobacco to aggregate exposure does not account for smoking’s effect on urinary arsenical levels. An NHANES 1999–2002 estimate of usual daily consumption of 16 cigarettes [58] and the amount of arsenic (0.0104 μg) in smoke generated from a reference cigarette [59] yields an exposure of 0.17 μg of arsenic per day. For an individual daily consuming two liters of drinking water containing arsenic at the current MCL (10 μg per l), the contribution of arsenic from cigarette smoking to aggregate exposure is nugatory (~0.8 % of total daily exposure). Hence, the association between smoking and higher levels of arsenicals in urine must reflect smoking-induced alterations that alter urinary clearance of arsenicals. Notably, dose–response relations for lung cancer relative risks are similar for inhaled or ingested iAs, suggesting that common metabolic and kinetic processes must determine the distribution and clearance of absorbed iAs or its metabolites that induce lung cancer [60]. The modifying effect of tobacco use on arsenic metabolism is an important public health issue because smoking and arsenic exposure have synergistic effects on lung and urinary bladder cancer and cardiovascular disease risk [61–63]. Similar to effects associated with active smoking, passive exposure to environmental tobacco smoke has also been associated with changes in SMI and the risk of urinary bladder cancer in individuals chronically exposed to iAs [64].

Seafood, including finfish and shellfish, can be significant dietary sources of inorganic or methylated arsenicals [65]. Analysis of dietary consumption data for U.S. populations found that total arsenic levels in urine were higher in individuals with seafood consumption within the last 24 h [66, 67]. In seafood consumers, the largest contributors to increased urinary total arsenic were arsenobetaine and dimethylated arsenic. Increased levels of urinary dimethylated arsenic may reflect degradation of complex organic arsenicals in seafoods [67]. We previously found that recent seafood consumption significantly increased urinary total arsenic levels but not increase USAs levels [1]. In the stepwise general linear model, seafood consumption significantly predicted urinary total arsenical but not USAs or NTAs concentrations. In the present study, the failure to detect a contribution of recent seafood consumption to levels of inorganic or methylated arsenicals in urine may reflect the low rate of recent seafood consumption among study participants (29 % within the last 48 h) or that the predominant species of arsenic in consumed seafood was neither a dimethylated arsenical nor a species that can be metabolized to dimethylated arsenical.

This analysis found that both biological and behavioral factors were significant predictors of absolute and relative levels of iAs, MMA, and DMA in urine. Because metabolism of iAs is linked to formation of methylated metabolites that likely mediate some of the adverse health effects associated with chronic exposure to iAs [6, 7], attempts to elucidate dose–response relations in population-based studies should consider potentially modifying effects of these factors. Accounting for these factors will improve assessment of risk associated with chronic exposure to iAs. Notably, in the multivariate regression model for the urinary concentrations of TiAs, MMA, DMA, and USAs practically all parameter estimates were statistically significant, suggesting that water source and consumption as well as a wide range of biological and behavioral factors influenced levels of these analytes in urine. In contrast, the multivariate regression models for %TiAs, %MMA, %DMA, and for the methylation indices PMI and SMI yielded a smaller set of parameter estimates that were statistically significant. Here, primarily biological and behavioral factors were the significant predictors of the response variables, suggesting that these factors modify the capacity to convert iAs to its methylated metabolites over a wide range of exposure conditions.

## Conclusions

Among study participants from a U.S. population with long histories of exposure to TAs from home tap water sources, we found that urinary levels of iAs and its mono- and di-methylated metabolites were predicted by age, gender, BMI, and smoking. A multivariate regression model of these data indicated that relative amounts of arsenical species in urine were also predicted by these biological and behavioral factors. Given the critical role of methylated arsenicals as mediators of the toxic and carcinogenic effects associated with exposure to iAs, this analysis underlines the need to evaluate potentially modifying effects of these factors in population-based studies of adverse health effects of this metalloid.

## Additional file

Additional file 1: Supplemental Information. (DOCX 512 kb)

## Abbreviations

AS3MT: arsenic (+3 oxidation state) methyltransferase
BMI: body mass index
DMA: dimethylated arsenic
DMA^V^: dimethylarsinic acid
iAs: inorganic arsenic
iAs^III^: arsenite
iAs^V^: arsenate
LOD: limit of detection
MCL: maximum contaminant level
MMA: monomethylated arsenic
MMA^V^: monomethylarsonic acid
NTAs: total arsenic in toenail samples
PMI: primary methylation index
SMI: secondary methylation index
TAs: total arsenic in home tap water
TiAs: sum of iAs^III^ and iAs^V^
USAs: sum of TiAs, MMA, and DMA
