# Supplementary material for: Biological and behavioral factors modify urinary arsenic metabolic profiles in a U.S. population
Source: Environ Health. 2016 May 26;15:62. doi: 10.1186/s12940-016-0144-x (PMC4880853; doi:10.1186/s12940-016-0144-x)
Supplement: Additional file 1: — Supplemental Information. (DOCX 512 kb) [file 12940_2016_144_MOESM1_ESM.docx]

**Additional Files**

**Biological and behavioral factors modify urinary arsenic metabolic**

**profiles in a U.S. population**

Edward E. Hudgens^1^, Zuzana Drobna^2^, Bin He^3^, X. C. Le^3^, Miroslav Styblo^2^,

John Rogers^4^, David J. Thomas^5^

1. Environmental Public Health Division, National Health and Environmental Effects Research Laboratory, Office of Research and Development, U.S. Environmental Protection Agency, Research Triangle Park, North Carolina 27709
2. Department of Nutrition, University of North Carolina at Chapel Hill, Chapel Hill, North Carolina 27599
3. b. Analytical and Environmental Toxicology, Department of Laboratory Medicine and Pathology, Faculty of Medicine and Dentistry, University of Alberta, Edmonton, Alberta, T6G 2G3 Canada
4. Westat, 1600 Research Boulevard, Rockville, MD 20850
5. Integrated Systems Toxicology Division, National Health and Environmental Effects Research Laboratory, Office of Research and Development, U.S. Environmental Protection Agency, Research Triangle Park, North Carolina 27709

**Table of Contents**

| **Section** | **Description** | **Pages** |
| --- | --- | --- |
| **1** | ***Demographic characteristics of study participants*** | 3 |
| **2** | ***Supplemental Information on Sampling Procedures and Analytical Methods*** | 3-8 |
|  | A. Tap water sample collection and assignment of home tap water total arsenic concentrations for study participants | 3 |
|  | B. Speciation of arsenicals in urine | 3-4 |
|  | C. Toenail As analysis | 4 |
|  | D. Urinary cotinine and creatinine measurements | 4-5 |
|  | E. AS3MT genotypic variation | 5-8 |
| **3** | ***Supplemental Information on Statistical Methods*** | 9-17 |
|  | 1. Imputation of missing and non-detect values | 9 |
|  | 1. Summary of missing values and non-detects | 9 |
|  | 1. Overview of the imputation process | 9-10 |
|  | 1. Imputation models for variables with missing values | 10-12 |
|  | 1. Results from the imputations process | 12-14 |
|  | 1. Overview | 12-13 |
|  | 1. Urinary arsenical concentrations | 13 |
|  | 1. Home tap water arsenic concentrations | 13-14 |
|  | 1. Toenail arsenic concentrations | 14 |
|  | B. Non-linear transformation of water arsenic concentration | 15-16 |
|  | i. Overview | 15 |
|  | ii. Procedure | 15-16 |
|  | C. Candidate variables for the stepwise regression | 16-17 |
|  | D. Comparison of urinary levels of arsenicals and incidence of non-detect values in Churchill County and NHANES survey data | 18-19 |

**Section 1 Demographic Characteristics of Study Participants**

**Demographic characteristics of study participants^1^**

| **Characteristics** | **Number (%)** |
| --- | --- |
| Gender  Male | 368 (41) |
| Female | 536 (59) |
| Age  45-49 | 120 (13) |
| 50-59 | 287 (32) |
| 60-69 | 242 (27) |
| 70-79 | 197(22) |
| 80-92 | 58 (6) |
| Self-reported smoking status  Nonsmoker | 752 (83) |
| Passive smoker | 34 (4) |
| Smoker | 118 (13) |

1. Census 2000 (U.S. Census Bureau, <http://factfinder2.census.gov>) reported that 31.6% and 34.3% of the population of Fallon and Churchill County, Nevada, respectively, was 45 years of age or older. For the U.S. population in 2000, 34.4% of the population was 45 years of age or older.

**Section 2. Supplemental Information on Sampling Procedures and Analytical Methods**

1. **Tap water sample collection and assignment of home tap water total arsenic concentrations for study participants**

At enrollment, study participants received 250 ml polypropylene sample bottles for collection of home tap water samples. These bottles were provided by the Nevada State Health Laboratory and were rinsed with nitric acid; residual nitric acid remained in each bottle. Participants were instructed to take a water sample at the cold water tap most often used as their drinking water source and to flush the inlet line for a least one minute before sample collection. Participants returned samples to the investigators on the day after collection. Samples were held at room temperature until transferred to the laboratory.

About 70% of study participants who used the Fallon municipal water system provided home tap water samples. For each of these participants, the measured home tap water total arsenic level was used for all subsequent data analysis. The mean total arsenic concentration in the home tap water samples provided by these participants was 89 µg per liter. For study participants who used the Fallon municipal water system but did not provide a home water sample, a home tap total arsenic concentration of 89 µg per liter was used in subsequent data analysis. For study participants who did not use the Fallon municipal water system, total arsenic concentration was determined by analysis of a home tap water sample. Some study participants who did not use the Fallon municipal water system had multiple sources for home tap water. In those cases, the mean home tap total arsenic concentration in these samples was used in subsequent data analysis. All study participants from a single household were assigned the same home tap total arsenic concentration.

**B. Speciated arsenicals in urine**

Concentrations of inorganic and methylated arsenicals in urine were determined by ion-pair chromatographic separation with hydride generation-atomic fluorescence detection [1]. The high performance liquid chromatography system consisted of a model 307 pump (Gilson, Middleton, WI), a model 7725i 6-port sample injector (Rheodyne, Rohnet Park, CA) with a 20 µl sample loop, and a reversed-phase C18 column (ODS-3, 150x4.6 mm, 3 µm particle size, Phenomenex, Torrance, CA). The mobile phase was 5mM tetrabutylammonium hydroxide in 3mM malonic acid (pH5.9) with 5% methanol at a flow rate of 1.2 ml per minute. Column temperature was maintained at 50^o^C. A hydride generation-atomic fluorescence detector (Model Excalibur 10.003, P.S. Analytical, Kent, UK) was used for detection of separated arsenicals. Analytical limits of detections (LODs) were 0.5 μg of arsenic per liter for arsenite (iAs^III^) and monomethylarsonic acid (MMA^V^) and 1 μg of arsenic per liter for arsenate (iAs^V^) and dimethylarsinic acid (DMA^V^). Standard reference material (CRM No. 18 human urine, National Institute of Environmental Studies, Japan) was used for quality control.

1. Le XC, Lu X, Ma M, Cullen WR, Aposhian HV, Zheng B. Speciation of key arsenic metabolic intermediates in human urine. Anal Chem 2000;72:5172–5177.

**C. Toenail arsenic analysis**

Toenail samples were cleaned and processed as previously described [1] and total arsenic concentrations were determined by instrumental neutron activation analysis (NAA) at the Nuclear Services Department of North Carolina State University, Raleigh, NC. Analytical accuracy for arsenic determination by NAA was confirmed using reference materials (CNRC-DORM2 dogfish muscle, CNRC-DOLT2 dogfish liver, Institute for National Measurement Standards, Ottawa, Ontario, Canada, and SRM RM-50 tuna, National Institute of Standards and Technology, Gaithersburg, MD) and were within 10% of the certified value.

1. Adair BM, Hudgens EE, Schmitt MT, Calderon RL, Thomas DJ. Total arsenic concentrations in toenails quantified by two techniques provide a useful biomarker of chronic arsenic exposure in drinking water. Environ Res. 2006;101:213-220.

**D. Urinary cotinine and creatinine measurements**

Urinary cotinine concentrations were determined by radioimmunoassay with a LOD of 2 ng of cotinine per ml of urine [1]. Cotinine concentrations were expressed both as measured and on a creatinine-corrected basis [2], providing log_10_-transformed creatinine-corrected cotinine concentrations. The multivariate regression model included log_10_-transformed cotinine and log_10_-transformed creatinine concentrations as predictors. Further details on cotinine and creatinine analysis of urine samples have been presented [3]. Creatinine concentrations in urine samples were determined on an Ortho-Clinical Diagnostics Model Vitros 950 analyzer (Ortho, Rochester, NY) in the McClendon Clinical Laboratories, Department of Pathology and Laboratory Medicine, School of Medicine, University of North Carolina at Chapel Hill (CLIA ID # 34D0655124).


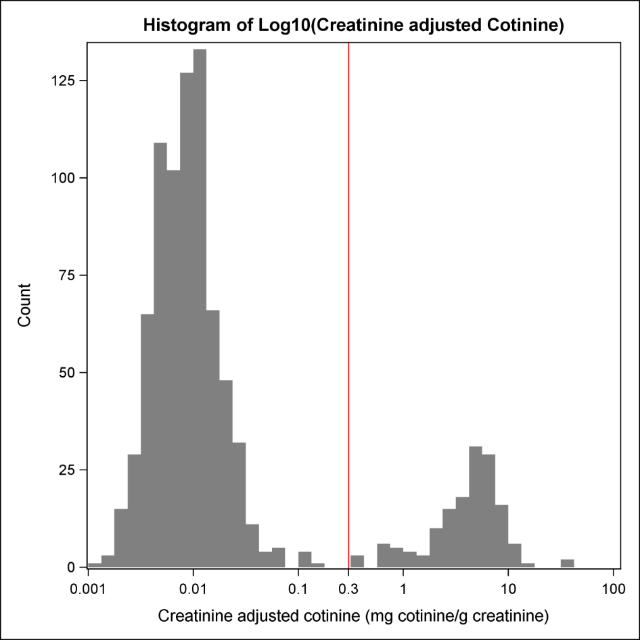
A histogram of creatinine-corrected urinary cotinine concentrations for study participants showed a bimodal distribution of values (Figure SI-1). Thus, for categorical analysis of the role of tobacco smoke exposure as a behavioral factor that may affect formation and urinary clearance of iAs and its methylated metabolites, we have designated study participants with creatinine-adjusted cotinine levels lower that 0.3 mg per g creatinine as non-smokers and study participants with levels at or above 0.3 mg per g creatinine as smokers.

1**.** Van Vunakis H, Gjika HB, Langone JJ. Radioimmunoassay for nicotine and cotinine. IARC Sci Publ. 1987; 81:317-330.

**Figure SI-1** – Distribution of creatinine-adjusted urinary cotinine concentrations.

## 2. Thompson SG, Barlow RD, Wald NJ, Van Vunakis H. How should urinary cotinine concentrations be adjusted for urinary creatinine concentration? Clin Chim Acta. 1990;187:289-295.

3. Calderon RL, Hudgens EE, Carty C, He B, Le XC, Rogers J, et al. Biological and behavioral factors modify biomarkers of arsenic exposure in a U.S. population. Environ Res. 2013;126:134-144.

**E. AS3MT Genotypic Variation**


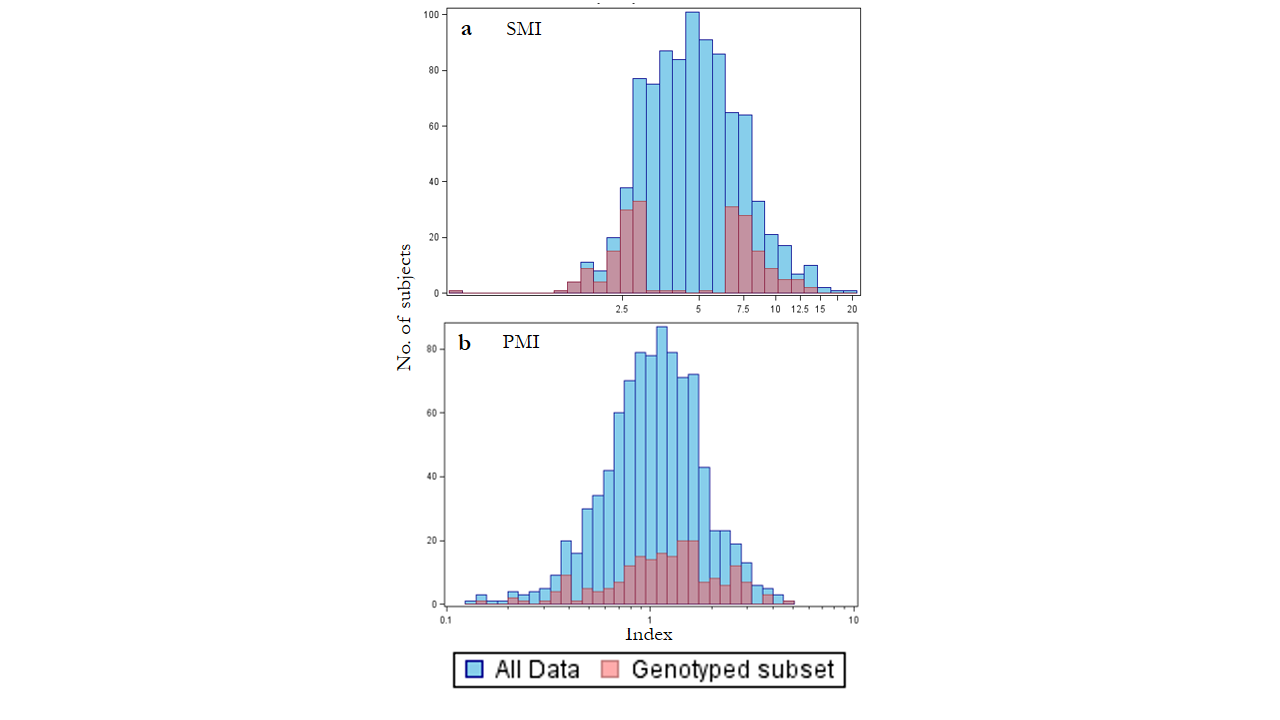
The *AS3MT* gene encodes the enzyme that catalyzes reactions that convert iAs into MMA and DMA [1]. Both intronic and exonic SNPs have been associated with altered arsenic methylation phenotype and variation in susceptibility to adverse health effects associated with chronic exposure to iAs [2, 3]. The effect of *AS3MT* SNP rs11191439 which substitutes a threonyl residue for a methionyl residue in position 287 on SMI is consistent with evidence linking altered kinetic properties of AS3MT to *AS3MT* genotype [4]. *AS3MT* SNP rs11191439 allelic frequency likely contributes to interpopulation and interindividual variation in arsenic methylation phenotype and in disease susceptibility [5]. An *AS3MT* haplotype associated with methylation status of AS3MT and other genes in chromosome band 10q24 may affect methylation phenotype through altered expression of *AS3MT* and surrounding genes [6]. Integrating *AS3MT* genotype and haplotype data into dose-response models may reduce uncertainty in assessing risk of chronic iAs exposure.

A pilot study examined relations between arsenical methylation phenotype and single nucleotide polymorphisms (SNPs) and variable number tandem repeats (VNTR) for *AS3MT*. For this analysis, 198 study participants selected primarily from the extremes of the SMI distribution were genotyped. Speciated arsenical analysis of urine samples from 904 study participants yielded data on concentrations of TiAs, MMA, and DMA. These data were used to calculate primary (PMI) and secondary (SMI) metabolic indices. For selection of a subset of samples for *AS3MT* genotyping, SMI values were sorted in ascending order. As shown in Figure SI-2a, the distribution of log_10_-transformed SMI values was approximately normal. From this distribution, 200 samples were selected for genotyping. The majority of these samples were taken from the extremes of the distribution with a few taken from the middle of the distribution. As shown in Figure SI-2b, selection on the basis of SMI values lead to a broad distribution of PMI values.

**Figure SI-2** – Distribution of methylation index values for samples selected for AS3MT genotyping. a). Distribution of selected samples sorted by secondary methylation index (SMI), b). Distribution of selected samples sorted by primary methylation index (PMI).

DNA was successfully isolated from 198 of the 200 samples selected for analysis. DNA was purified from 7 to 10 milliliters of citrate-preserved venous blood using a QIAamp DNA Blood Mini Kit (QIAGEN, Valencia, CA). Isolated DNA was stored at -80^o^C. Functional single nucleotide polymorphisms (SNPs) in AS3MT that have been linked to differences in iAs metabolism or in susceptibility to iAs toxicity were determined in the UNC Mammalian Genotyping Core, using functionally tested TaqMan assays (rs35232887, rs11191439, and rs34556438) and a custom Taqman genotyping assay (rs11191453) purchased from AB Applied Biosystems (Foster City, CA). DNA samples of plasmids (p91023B/hAS3MT/Arg173Trp, pRSET/hAS3MT/Met287Thr, p91023B/hAS3MT/Thr306Ile) carrying three most common exonic SNPs were used as positive controls. P91023B/hAS3MT’s constructs were kindly provided by Dr. Richard Weinshilboum (Mayo Clinic College of Medicine, Rochester, MN). Variable number of tandem repeats (VNTR) that affect AS3MT expression [7] were analyzed by sequencing of the corresponding 5’-UTR region after PCR amplification PCR products amplified by using VNTR primers were also used to evaluate another SNP (rs17881215) located just 37 bases upstream from VNTR structure. Table SI-1 provides information on polymorphisms examined in this study as well as the primers used for amplification.

**Table SI-1** – Primers used for amplification of AS3MT SNPs

| **SNP** | **Primers** | **Polymorphism** |
| --- | --- | --- |
| rs11191439 | forward: 5'-GGAGTCTCATTGAGGGATAC-3'  reverse: 5'-GTGAACTATGATTGTGCTACTG-3' | Met287Thr |
| rs11191453 | forward: 5'-CACCACACCCAGCTAA-3'  reverse: 5'-CTTGGGCAGAGCATTGA-3' | Intronic |
| rs17881215  and VNTR | forward: 5'-GATCATTATATAGGTGAGTGTTCATTTA-3' reverse: 5'-AGCGGGAAAGTTAGTTGAAA-3' | Intronic |


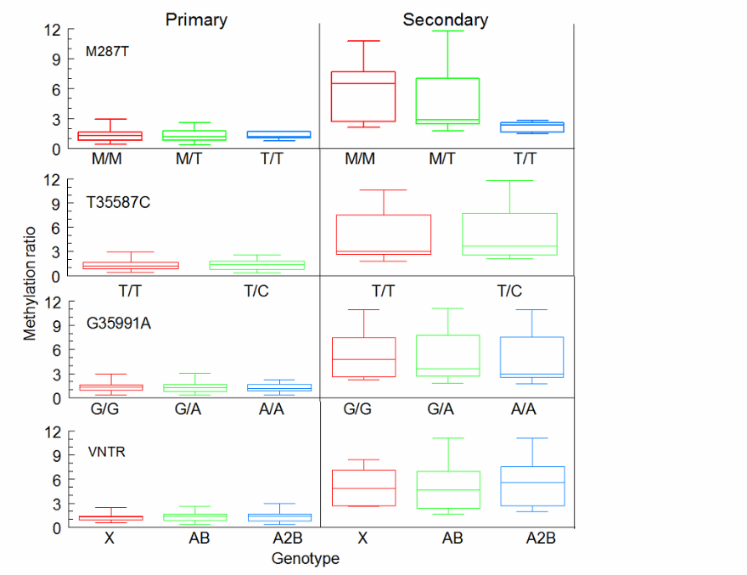


Figure SI-3 shows relations between methylation indices and *AS3MT* SNPs and VNTRs. The SNPs were rs11191439 which replaces the methionyl residue in position 287 with a threonyl residue (M287T) and two intronic SNPs, rs11191453 (T35587C - T>C) and rs10748835 (G35991A – G>A). VNTRs from *AS3MT*’s 5′-untranslated region which may affect gene expression were examined [7]. The M287T substitution strongly affected SMI with lowest values found in homozygotes but had no effect on PMI. For the M287T variant, a Kruskal-Wallis ANOVA on ranks found differences in median SMI values for M/M, M/T, and T/T (P≤0.001). Pairwise comparison of SMI values by Dunn’s method found a significant difference (P<0.05) between M/M and M/T and between M/M and T/T but no significant difference between M/T and T/T. In contrast, no genotype for either intronic SNP and for VNTR was significantly associated with values of either methylation index.

**Figure SI-3** – Effect of *AS3MT* polymorphisms on the Primary and Secondary methylation indices in 198 study participants selected for genotyping on the basis of Secondary methylation index values.

1. Thomas DJ, Li J, Waters SB, Xing W, Adair BM, Drobna Z, et al. Arsenic (+3 oxidation state) methyltransferase and the methylation of arsenicals. Exp Biol Med (Maywood). 2007;232:3-13.

2. Pierce BL, Tong L, Argos M, Gao J, Farzana J, Roy S, et al. Arsenic metabolism efficiency has a causal role in arsenic toxicity: Mendelian randomization and gene-environment interaction. Int J Epidemiol. 2013;42:1862-1871.

3. Antonelli R, Shao K, Thomas DJ, Sams R 2nd, Cowden J. *AS3MT*, *GSTO*, and *PNP* polymorphisms: impact on arsenic methylation and implications for disease susceptibility. Environ Res. 2014;132:156-167.

4. Ding L, Saunders RJ, Drobna Z, Walton FS, Xun P, Thomas DJ, et al. Methylation of arsenic by recombinant human wild-type arsenic (+3 oxidation state) methyltransferase and its methionine 287 threonine (M287T) polymorph: Role of glutathione. Toxicol Appl Pharmacol. 2012;264:121-130.

5. Agusa T, Fujihara J, Takeshita H, Iwata H. Individual variations in inorganic arsenic metabolism associated with AS3MT genetic polymorphisms. Int J Mol Sci. 2011;12:2351-2382.

6. Engstrom KS, Hossain MB, Lauss M, Ahmed S, Raqib R, Vahter M, et al. Efficient arsenic metabolism—the AS3MT haplotype is associated with DNA methylation and expression of multiple genes around AS3MT. PLoS One. 2013;8:e537327.

7. Wood TC, Salavagionne OE, Mukherjee B, Wang L, Klumpp AF, Thomae BA, et al. Human arsenic methyltransferase (AS3MT) pharmacogenetics: gene resequencing and functional genomics studies. J Biol Chem. 2006;281:7364-7373.

**Section 3. Supplemental Information on Statistical Methods**

## A. Imputation of missing and non-detect values

There were multiple instances in which data were unavailable or missing. In some cases, an individual datum was unavailable because the concentration of an analyte was below the analytical limit of detection. These data are referred to as non-detects. In other cases, an individual datum was missing because it was not collected during the study. These data are assumed to be missing-at-random. The following sections describe procedures used to impute values for non-detect or missing-at-random data.

1. **Summary of missing values and non-detects**

Table SI-2 summarizes the number of non-detect or missing values for variables.

**Table SI-2** Summary of missing and non-detect values

| **Variable** | **Number missing** | **Number**  **non-detects** |
| --- | --- | --- |
| Urinary DMA concentration | 0 | 46 |
| Urinary MMA concentration | 0 | 217 |
| Urinary iAs^III^ concentration | 0 | 289 |
| Urinary As^V^ concentration | 0 | 583 |
| Home tap water As concentration | 0 | 21 |
| Toenail As | 59 | 52 |
| Urinary cotinine concentration | 1 | 43^a^ |
| Urinary creatinine concentration | 1 | 0 |
| BMI | 1 | 0 |

1. The urine cotinine concentrations include 43 non-detects recorded as zero. No detection limit was provided. Based on the distribution of the log transformed non-zero concentrations, the zeroes were replaced by 1.0 ng/ml urine, half of the smallest non-zero value and were not otherwise imputed.

For these analyses, the missing values were handled using multiple imputation. Multiple imputation involves creating multiple copies of the data set (in this case 20 copies), and imputing the missing values in each data set. The imputation process replaces the missing values with plausible substitute values that are consistent with the detection limits and consistent with the relationships between variables. Each data set has a different set of imputed values. Using multiple imputation allows fitting models while using all records and calculation of appropriate standard errors that account for the fact that the imputed missing and non-detect values are uncertain.

1. **Overview of the imputation process**

A Bayesian statistical model was used to impute the missing values in all variables in the same model run. The model includes equations that describe relationships among variables in the data set. When there are non-detects (or the missing values are not missing-at-random), the imputation process also requires equations to predict the probability of a missing or non-detect value.

Imputation equations were selected in a three step process:

Step 1 - The SAS Bayesian MCMC procedure was run to impute all missing values for the variables in Table SI-2 assuming linear relationships among the variables, without any interactions.

Step 2 - Using 10 imputed datasets from step 1, stepwise regression was used to select interactions for predicting each of the variables with missing values. Interactions were included in the final imputation model if they were selected in 5 of the 10 imputed datasets. The SAS GLMSELECT stepwise procedure used default settings, except that two-way interactions were included only if both main effects in the interaction were previously included in the model.

Step 3 - The SAS Bayesian MCMC procedure was run to impute all missing values for the variables in Table SI-2 including linear terms and selected interactions, 20 imputed datasets were created for analysis.

1. **Imputation Models for all Variables with Missing Values**

Table SI-3 lists (in order of use) variables used to impute missing values. Variables highlighted in yellow had missing values that were imputed. For variables with missing values, all variables in previous rows were used as predictor variables, unless otherwise noted. For categorical variables represented by several dummy variables, the names of the dummy variables are shown in square parentheses.

**Table SI-3** Variables used in imputation of missing values

| **Variable Type** | **Variable** | **Description** |
| --- | --- | --- |
| Variables with no missing values, used as predictors in one of the imputation models | CityWater | An indicator of whether the tap water came from the city water system (1) or a local well (0). 18 of 250 values reported as city water were judged to be outliers and were assumed to be from well water. CityWater was used as a predictor for only the arsenic concentration in home tap water. |
|  | CotSmoker [CS1, CS2] | Smoking status based on a combination of urine cotinine and reported smoking status (non-smoker, passive, active smoker) Cotsmoker was used as a predictor for only Log(Cotinine) |
| Variables with no missing values, used as predictors in all imputation models below | Drinksource [DTap, DTreat] | Primary source of drinking water (Tap, treated, or bottled water) |
|  | TrtFilt | Treated tap water with a filter for treatment (1), otherwise 0 |
|  | FSF48 | Consumption of fish or shellfish within last 48 hours; (1-Yes, 0=No) |
|  | Female | Gender (1=Female, 0 = Male) |
|  | LogAge | Log10(age in years) |
|  | QTT | Log10(daily consumption of tap & treated water); 0 replaced by 0.06 |
|  | QTap | Log10(daily tap water consumption); 0 replaced by 0.06 |
|  | Qtot | Log10(daily water consumption from all sources) |
|  | LogTotAs | Log10(Total urinary arsenic) |
| Variables with missing values to be imputed | LogCot | Log10(Cotinine) (Cotinine values of 0 were set to 1) |
|  | LogBMI | Log10(BMI) |
|  | LogCreat | Log10(Creatinine) |
|  | LogWaterAs | Log10(Arsenic concentration in home tap water) |
|  | LogDMA | Log10(urine DMA^V^ concentration) |
|  | LogMMA | Log10(urine MMA^V^ concentration) |
|  | LogAsIII | Log10(urine As^III^ concentration) |
|  | LogAsV | Log10(urine As^V^ concentration) |
|  | LogNailAs | Log10(toenail arsenic concentration) |

Stepwise selection procedures were used to select interactions that were included in the imputation model. Table SI-4 shows the interactions included in each model and the assumed model for missing values. Urine arsenic species were classified as non-detects based on the results from three measurements on the same sample. If any of the three measurements was below the nominal detection limit, then the sample was classified as a non-detect. The probability that a sample is classified as a non-detect was approximated by a logistic function.

**Table SI-4** Interactions and missing value models used for each imputed variable

| **Dependent Variable** | **Interactions and special situations** | **Missing value model** |
| --- | --- | --- |
| LogCot | Included CS1 and CS2 (dummy variables for CotSmoker) as predictors | None, assumed missing at random |
| LogBMI |  | None, assumed missing at random |
| LogCreat |  | None, assumed missing at random |
| LogWaterAs | Concentrations from city water samples were modeled using just the mean; multiple predictors were used to predict water concentrations from well samples. The equations used different standard deviations for the well samples and city water samples.  Interactions: QTT*LogCreat and QTT* LogTotAs | Non-detect if LogWaterAs < Log(WaterAsDL) ^a^ |
| LogDMA | Interactions: FSF48* LogTotAs, LogTotAs*logWaterAs, FSF48*logCreat, FSF48*QTT, LogTotAs *QTap | Probability of non-detect = Logistic (LogDMA) ^b^ |
| LogMMA |  | Probability of non-detect = Logistic (LogMMA) ^b^ |
| LogAsIII | Interaction: FSF48* LogTotAs, | Probability of non-detect = Logistic(LogAsIII) ^b^ |
| LogAsV |  | Probability of non-detect = Logistic (LogAsV) ^b^ |
| LogNailAs |  | Non-detect if LogNailAs < Log(NailAsDL) ^a^  Otherwise missing at random if no toenail sample |

a. Specifying a zero probability for a non-detect if the simulated concentration was above the detection limit resulting in numerical problems. This was handled by specifying a very small non-zero probability (0.000001) and excluding the few simulated values for which the simulated non-detect concentration was above the detection limit.

b. Logistic(X) means a logistic model with two parameters, an intercept and X as a predictor.

We saved 1000 simulated parameter values and imputed values. In the final imputation model results, all parameters had an effective degrees of freedom of at least 200; 20 sets of imputed values were selected using a systematic sample.

1. **Results from the imputation process**
2. **Overview**

Results obtained by imputation were examined to assure that distributions of imputed values were consistent with distributions of the detected measurements, to verify that imputed results were reasonable, to check for convergence, and to evaluate whether parameter estimates were reasonable. Notably, imputed results met these criteria.

1.
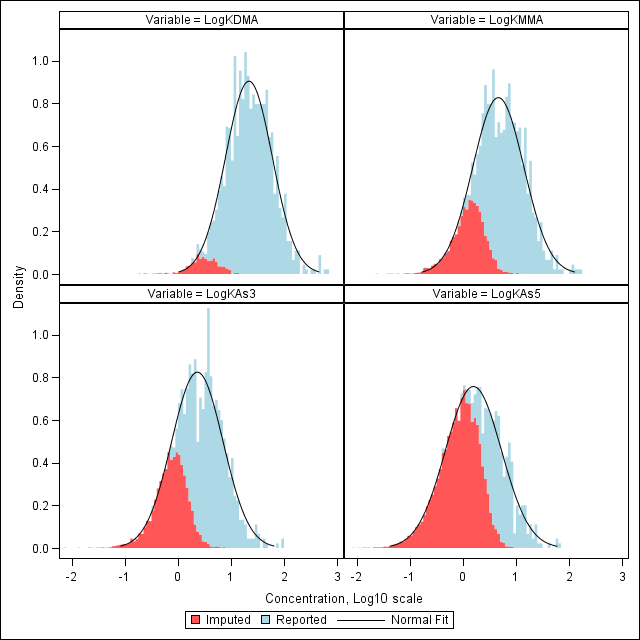
**Urinary arsenical concentrations**

A logit function was used to describe the probability that a measurement of an arsenical in urine would be classified as a non-detect. As a result, there is a smooth transition from non-detected values to detected values as the concentration increases. For each of the urine arsenic species, Figure SI-4 shows the distributions of all values in the imputed variables.

1. **Home tap water total arsenic concentrations**

The detection limit for total arsenic concentration was identical for all samples. Hence, all imputed values for non-detects are all less that the detection limit.
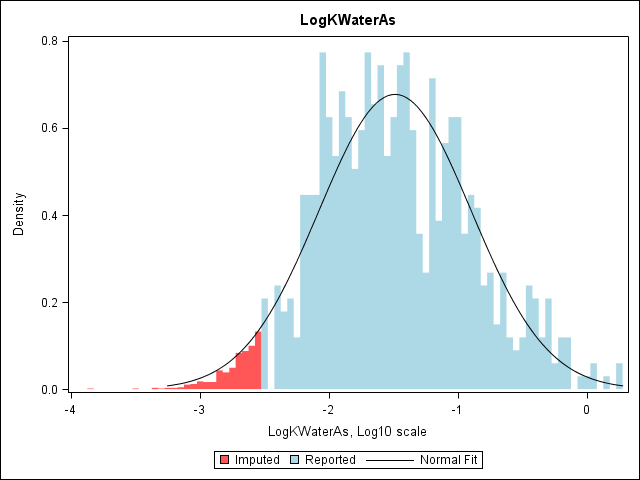
Figure SI-5 shows the distribution of the total arsenic concentrations is tap water from sources other than the Fallon municipal water supply.

**Figure SI-4** - Distributions of imputed (K) values for concentrations of urinary arsenicals. a.) inorganic arsenite, As3, b.) inorganic arsenate, As5, c.) monomethylated arsenic (MMA), and d.) dimethylated arsenic (DMA). Figure shows a histogram of all values with imputed values shaded in red and detected concentrations shaded in blue as well as the approximate lognormal distribution assumed for all values (as a blue line). Concentrations of urinary arsenicals expressed in parts per billion of arsenic.

1. **Toenail arsenic concentrations**

The detection limits for arsenic concentration varied among toenails as a function of the mass of the sample. Thus, imputed values for toenail arsenic, although less than the detection limit, are spread over a range. In addition, for respondents who did not provide a home tap water sample for determination of total arsenic it was assumed that the concentration of arsenic in the water sample was missing at random. Hence, imputed values for toenail arsenic ranged over the distribution of observed values (Figure SI-6).

**Figure SI-5** - Distributions of imputed (K) values for concentrations of total arsenic in home tap water from wells. Figure shows a histogram of all values with imputed values shaded red and detected values shaded in blue as well as the approximate lognormal distribution assumed for all values (as a blue line). Concentrations of total arsenic in home tap water expressed in parts per million of arsenic.


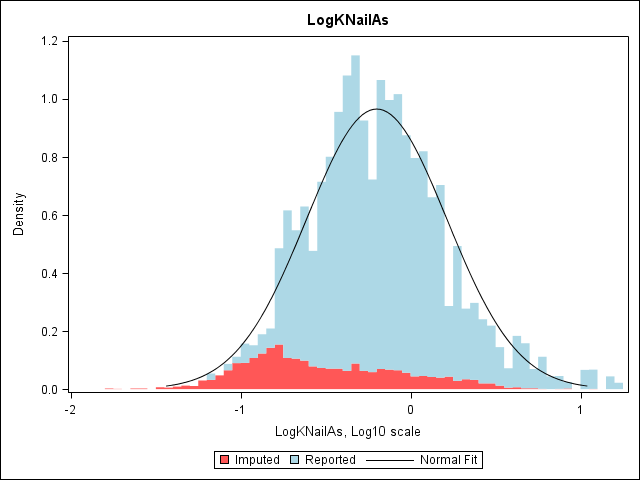


**Figure SI-6 -** Distributions of imputed (K) values for concentrations of arsenic in toenails. Figure shows a histogram of all values with imputed values shaded in red and detected non-missing values shaded in blue, as well as the approximate lognormal distribution assumed for all values (as a blue line). Concentrations of arsenic in toenails expressed in parts per million of arsenic

**B. Non-linear Transformation of Water Arsenic Concentration**

**i. Overview**

Stepwise regression used to predict log-transformed summed urinary concentrations of inorganic arsenic and its methylated metabolites selected a quadratic relationship for log-transformed home tap water arsenic concentration. Because transformation of one predictor can affect parameter estimates of other predictors, multiple models were fitted to determine which continuous log-transformed variables (home tap water consumption, summed urinary concentrations of inorganic arsenic and its methylated metabolites, urinary creatinine concentration, and home tap water arsenic concentration) required transformation.

**ii. Procedure**

The SAS TRANSERG procedure was used to fit spline (i.e., smooth non-linear) functions in a regression model. Spline functions can be applied to multiple continuous variables in the same model if at least one continuous variable is not transformed. Multiple models were fitted using different dependent variables and different sets of variables to be transformed. The TRANSERG output includes a plot of the estimated transformation for each continuous variable.

This analysis indicated that:

- Log-transformed urinary creatinine concentration did not require additional transformation, regardless of whether the other variables are transformed,
- Minimal (if any) transformation could be used for home tap water consumption and summed urinary concentrations of inorganic arsenic and its methylated metabolites, and
- Log-transformed home tap water arsenic concentration should be transformed, particularly when predicting log-transformed summed urinary concentrations of inorganic arsenic and its methylated metabolites. Estimated transformation for log-transformed home tap water arsenic concentration depended on whether home tap water arsenic concentration or summed urinary concentrations of inorganic arsenic and its methylated metabolites was the dependent variable.

Based on the estimated transformation of log-transformed home tap water arsenic concentration when predicting summed urinary concentrations of inorganic arsenic and its methylated metabolites, the following monotonic transformation was assumed:

$${Log}_{10}\left( C+Water As Concentration \right)$$

To evaluate this assumed transformation, non-linear regression models were fitted predicting log-transformed summed urinary concentrations of inorganic arsenic and its methylated metabolites as a function of transformed home tap water arsenic concentration from the equation above, log-transformed urinary creatinine consumption home tap water consumption, and other categorical variables. The parameter estimates for the concentration offset, C, were consistent across models (ranging from 0.0154 to 0.0164) and generally significantly different from zero (p < 0.055). When the square of log-transformed home tap water arsenic concentration was included in the model, it was not significant; suggesting that the non-linear function described above provides a consistent explanation for the quadratic term identified in stepwise regressions.

**C. Candidate Variables for the Stepwise Regression**

The following is a list of the candidate variables used in stepwise selection for models predicting log-transformed TiAs, MMA, DMA, USAs, SMI, PMI, and logit transformed percentages of DMA, MMA, and iAs. The selected variables were used to construct the final model fit to these variables.

- Log_10_-transformed imputed TAs (as µg/l)
- Log_10_-transformed imputed TAs squared (to assess non-linearity)
- Log_10_-transformed imputed urine creatinine (as mg/dl)
- Log_10_-transformed BMI
- Gender (1 = Female, 0 = Male)
- Log_10_-transformed Age (in years)
- An indicator of alcohol consumption in the past 24 hours
- Log_10_-transformed imputed urine cotinine (ng/ml)
- Race (White versus non-white)
- Smoker versus non-smoker defined from creatinine adjusted cotinine concentration
- Consumption of fish or shellfish on the past 48 hours (Yes/No)
- Consumption of fish in the past 48 hours (Yes/No)
- Consumption of Seafood in the past 48 hours
- Number of times fish and seafood is consumed in a week (as a continuous variable)
- Drinking water source is treated using filtration (Yes/No)
- Drinking water source is treated using reverse osmosis (Yes/No)
- Drinking water source is treated using a softener (Yes/No)
- Drinking water source is treated using another method (Yes/No)
- Drinking water source = tap water (Yes/No)
- Drinking water source = treated water (Yes/No)
- Drinking water source = bottled water (Yes/No)
- Both drinking water source and cooking water source = bottled water (Yes/No)
- Drinking water source (Categorical: Tap, Treated, Bottled)
- Cooking water source (Categorical: Tap, Treated, Bottled)
- Drinking water source for coffee or tea (Categorical: Don’t make hot drinks, Tap, Treated, Bottled)
- Drinking water source for juice or cold drinks (Categorical: Don’t make cold drinks, Tap, Treated, Bottled)
- Log_10_(daily consumption of tap water (treated or untreated) in liters, 0 replaced by 0.06) (QTT)
- Log_10_(daily consumption of untreated tap water in liters (0 replaced by 0.06))
- Log_10_(daily water consumption from all sources in liters)
- Log_10_(daily consumption of treated tap water in liters (0 replaced by 0.06))

Respondents were asked to specify the number of ounces of water they consumed daily from three sources, untreated tap water, treated tap water, or bottled water, and at three locations, home, work, and elsewhere. Totals for untreated tap water and treated tap water were the corresponding sum across the three locations. Total water consumption was the sum across locations and sources. Occasional missing values were treated as zeroes. For calculating log_10_ transformed water consumption, zeroes were replaced by 2 ounces, ounces were converted to liters and the result was log_10_ transformed.

**D. Comparison of urinary levels of arsenicals and incidence of non-detect values in Churchill County and NHANES survey data**

Table SI-5 shows geometric mean concentrations and mean detection limits for urinary arsenicals and percentages of non-detect observations among Churchill County study participants and corresponding values calculated from age-, gender-, and race-matched NHANES survey data for the period of 2002 to 2012. Survival analysis was used to estimate mean log_10_-transformed concentrations of urinary arsenicals in the NHANES data from which the geometric mean was calculated. NHANES data was then reweighted to represent the demographic distribution of the Churchill County study population, using using gender, race, and age groups. Statistics for the NHANES survey used these revised weights. Because detection limits for analytes in NHANES survey data varied over the years, the table shows average values Detection limits for analytes in the Churchill County study were similar to those for NHANES survey data. Higher geometric mean concentrations of arsenicals in urine of Churchill County study participants were reflected in ratios of concentrations above one and in the lower percentages of non-detect values.

| **Arsenical** | **Geometric mean (** **µg/l )** | | | **Mean detection limit**  **(** **µg/l )** | | **% Non-detects** | |
| --- | --- | --- | --- | --- | --- | --- | --- |
|  | **Churchill County** | **NHANES** | **Churchill County:NHANES Ratio** | **Churchill County^a^** | **NHANES** | **Churchill County^a^** | **NHANES** |
| Total As | 37.35 | 9.65 | 3.87 |  | 0.99 | 0.0 | 1.0 |
| iAs^III^ | 2.39 | 0.21 | 11.19 | 0.50 | 1.08 | 32.0 | 92.8 |
| iAs^V^ | 1.66 | 0.16 | 10.65 | 1.00 | 0.98 | 64.5 | 97.2 |
| MMA^V^ | 4.65 | 0.59 | 7.92 | 0.50 | 0.89 | 24.0 | 70.3 |
| DMA^V^ | 21.70 | 3.88 | 5.60 | 1.00 | 1.72 | 5.1 | 17.0 |

**Table SI-5.** Geometric mean, mean detection limit, and percentage of non-detect measurements for various arsenic species for the study population in Churchill County Nevada and a population with similar age, gender, and race distribution from the NHANES data.

a. No total arsenic measurements were below the detection limit. Shown is the nominal detection limit for each arsenic species. If any of three measurements were less than the nominal detection limit, the concentration in the sample was reported as a non-detect. Otherwise the average of the three measurements was used.
